# A Practical Treatise on Medical Diagnosis

**Published:** 1900-07

**Authors:** 


A Practical Treatise on Medical Diagnosis. For Students and Physicians. By John H. Musser, M. D., Professor of Clinical Medicine in the University of Pennsylvania, etc. Third Edition, Revised and Enlarged. Illustrated with 253 Woodcuts and 48 Colored Plates. 1082 pages. Price, $6. Lea Brothers & Co., Philadelphia and New York. 1899.

In 1894 the author issued the first edition of the above work. Its popularity forced a second issue in 1896, and three years later the third edition was called for, that being the present. Every page has been closely reviewed and revised, and all of the recent advances in methods of diagnosis have been added. Every up-to-date physician must live more in his laboratory than has been demanded of him heretofore, and no work with which the writer is familiar combines more comprehensively and practically the modern methods of medical diagnosis than that of Musser. The arrangement of the subject matter is most excellent and easy.

Part I is devoted to general diagnosis, including general observations,—the data obtained by inquiry and observation, by the morbid processes and their symptomatology, as applied to the different diseases.

Part II is devoted to special diagnosis, including the diseases of the nose, larynx, lungs, pluræ, heart, blood vessels, mediastinum, mouth, fauces, pharynx, esophagus, stomach, intestines, peritoneum, liver, spleen, pancreas, kidneys, and nervous symptoms. There are twenty-three chapters in Part I, and eight in Part II. T. J. B.

